# Increasing Incidence of *Geomyces destructans* Fungus in Bats from the Czech Republic and Slovakia

**DOI:** 10.1371/journal.pone.0013853

**Published:** 2010-11-05

**Authors:** Natália Martínková, Peter Bačkor, Tomáš Bartonička, Pavla Blažková, Jaroslav Červený, Lukáš Falteisek, Jiří Gaisler, Vladimír Hanzal, Daniel Horáček, Zdeněk Hubálek, Helena Jahelková, Miroslav Kolařík, L'uboš Korytár, Alena Kubátová, Blanka Lehotská, Roman Lehotský, Radek K. Lučan, Ondřej Májek, Jan Matějů, Zdeněk Řehák, Jiří Šafář, Přemysl Tájek, Emil Tkadlec, Marcel Uhrin, Josef Wagner, Dita Weinfurtová, Jan Zima, Jan Zukal, Ivan Horáček

**Affiliations:** 1 Institute of Vertebrate Biology, Academy of Sciences of the Czech Republic, Brno, Czech Republic; 2 Institute of Biostatistics and Analyses, Masaryk University, Brno, Czech Republic; 3 Department of Biology and Ecology, Matej Bel University, Banská Bystrica, Slovakia; 4 Department of Botany and Zoology, Masaryk University, Brno, Czech Republic; 5 Agency for Nature Conservation and Landscape Protection of the Czech Republic, Mariánské Lázně, Czech Republic; 6 Department of Zoology, University of South Bohemia, České Budějovice, Czech Republic; 7 Department of Forest Protection and Game Management, Czech University of Life Sciences Prague, Praha, Czech Republic; 8 Department of Zoology, Faculty of Science, Charles University in Prague, Praha, Czech Republic; 9 Agency for Nature Conservation and Landscape Protection of the Czech Republic, Praha, Czech Republic; 10 Sametová 721/18, Liberec, Czech Republic; 11 Department of Botany, Faculty of Science, Charles University in Prague, Praha, Czech Republic; 12 University of Veterinary Medicine and Pharmacy, Košice, Slovakia; 13 Department of Landscape Ecology, Comenius University, Bratislava, Slovakia; 14 Miniopterus - Principal Organization of the Slovak Union for Nature and Landscape Conservators, Bratislava, Slovakia; 15 Agency for Nature Conservation and Landscape Protection of the Czech Republic, Karlovy Vary, Czech Republic; 16 Agency of Nature Conservation and Landsape Protection of the Czech Republic, Olomouc, Czech Republic; 17 Department of Ecology and Environmental Science, Palacky University, Olomouc, Czech Republic; 18 Institute of Biology and Ecology, P. J. Šafárik University, Košice, Slovakia; 19 ZO ČSS 7-01 ORCUS Bohumín, Czech Speleological Society, Bohumín, Czech Republic; University of Sydney, Australia

## Abstract

**Background:**

White-nose syndrome is a disease of hibernating insectivorous bats associated with the fungus *Geomyces destructans*. It first appeared in North America in 2006, where over a million bats died since then. In Europe, *G. destructans* was first identified in France in 2009. Its distribution, infection dynamics, and effects on hibernating bats in Europe are largely unknown.

**Methodology/Principal Findings:**

We screened hibernacula in the Czech Republic and Slovakia for the presence of the fungus during the winter seasons of 2008/2009 and 2009/2010. In winter 2009/2010, we found infected bats in 76 out of 98 surveyed sites, in which the majority had been previously negative. A photographic record of over 6000 hibernating bats, taken since 1994, revealed bats with fungal growths since 1995; however, the incidence of such bats increased in *Myotis myotis* from 2% in 2007 to 14% by 2010. Microscopic, cultivation and molecular genetic evaluations confirmed the identity of the recently sampled fungus as *G. destructans*, and demonstrated its continuous distribution in the studied area. At the end of the hibernation season we recorded pathologic changes in the skin of the affected bats, from which the fungus was isolated. We registered no mass mortality caused by the fungus, and the recorded population decline in the last two years of the most affected species, *M. myotis*, is within the population trend prediction interval.

**Conclusions/Significance:**

*G. destructans* was found to be widespread in the Czech Republic and Slovakia, with an epizootic incidence in bats during the most recent years. Further development of the situation urgently requires a detailed pan-European monitoring scheme.

## Introduction

White-nose syndrome (WNS) is an emerging infectious disease, affecting hibernating insectivorous bats [1]. Since its first known appearance in 2006, WNS has spread with each year into the underground hibernacula in the USA and Canada, and over one million deaths within populations of several bat species have been attributed to the disease [1], [2]. The decline is severe enough to warrant a prediction that once common *Myotis lucifugus* might become locally extinct in less than two decades [2].

The most likely infectious agent of WNS is the newly described fungal species *Geomyces destructans* Blehert & Gargas, 2009 [3]. It is psychrophilic, and does not grow at temperatures higher than about 20°C. During hibernation, when the body temperature of bats drops to the ambient temperatures of the underground hibernacula, the fungal mycelia can grow upon the skin surfaces of these animals [1], [3]. The fungus invades the hair follicles and associated glands, or it breaks the epidermis of naked skin on the ears, muzzle, and wing membranes [1], [4]. The specific etiology of the fungal infection is unknown, but the bats awaken from hibernation. As arousal from hibernation is energetically demanding, it is believed that WNS leads to a more rapid disappearance of fat reserves [1], [4], [5], [6], deteriorating the body condition, and often to increased mortality due to starvation [7]. The bats prematurely emerge from the hibernacula and attempt to forage, which in winter conditions causes frostbite and subsequent necrosis of the wing membranes [8]. The fungal lesions co-infected with Gram-negative bacteria exhibit necrosis and pustules [5], furthering wing membrane damage and compromising flight abilities. Although not all of the details of the epizootics are fully understood [4], [5], [7], [8], [9], [10], it is widely accepted that WNS poses a severe threat for the bat populations in North America [2], [11]. The threat is likely to increase in the future, leading to local extinctions of the bats [2], and suggesting altering ecosystem dynamics [7], [10].

The problem may expand onto one on a global scale, as *G. destructans* was reported in France in March 2009 [12], and from Germany, Switzerland, and Hungary in the winter of 2008/2009 [13]. Here, we show that the occurrence of *G. destructans* in Europe is not episodic, but it is locally widespread and could be associated with skin lesions. We believe that *G. destructans* has been present historically within Europe, but that the epizootic is currently (re-)starting, with marked local differences in both the intensity and the dynamics of the disease.

## Results

### Historical Record of Geomycosis

**Figure 1:**
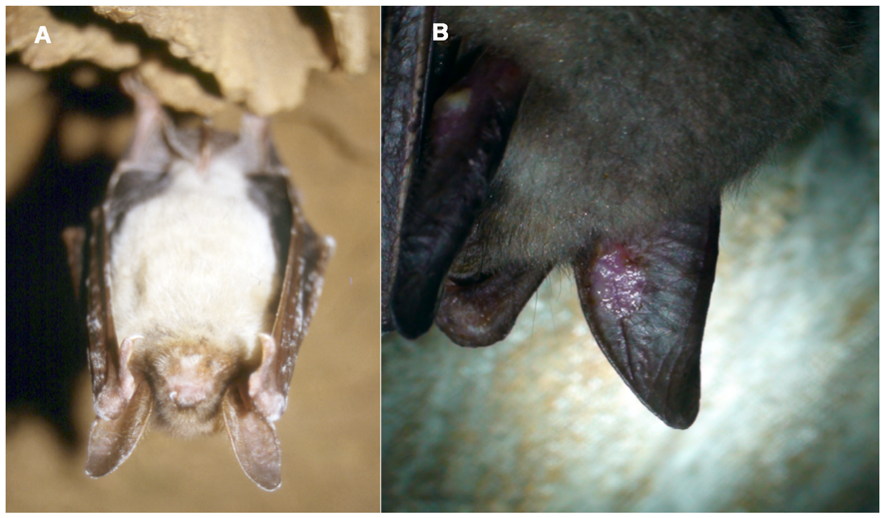
White-nose syndrome symptoms in the Czech Republic and Slovakia. (A) Hibernating *M. myotis* in the Javoříčské Caves, Czech Republic, photographed on 25 January 1997. Fungal growth was not identified. (Photo by Jiří Šafář) (B) Skin lesions on *M. myotis* from the Malá Amerika Mines, Karlštejn, Czech Republic, photographed on 16 March 2010. *G. destructans*, isolate number CCF3942, was isolated from the sample taken from the lesion. (Photo by Ivan Horáček).

The compilation of photographs of hibernating bats revealed a white patch on the muzzle of a *M. myotis* individual on March 4, 1995 from the Zbojnícka Cave, Malé Karpaty, Slovakia (Table 1). A photograph from January 25, 1997 from the Javoříčské Caves in northern Moravia, Czech Republic depicts a *M. myotis* individual with a fungal growth typical of the *G. destructans* infection (Fig. 1A). Further records show sporadic images of randomly photographed affected *M. myotis* until 2007/2008, when the incidence of bats with white patches started to increase in several species (Table 1).

**Table 1:** Summary of WNS-suspect bats from those photographed randomly (number of all photographed bats/number of photographed WNS-suspect bats) during the periods 1994–1998 and 2003–2010.

Winter period	1994/95	1995/96	1996/97	1997/98	2003/04	2004/05	2005/06	2006/07	2007/08	2008/09	2009/10
Species											
*R. hipposideros*	-	3/0	-	2/0	-	4/0	11/0	26/0	82/0	45/0	153/15
*M. bechsteinii*	-	-	-	-	1/0	2/0	-	-	3/0	3/0	8/0
*M. brandtii*	-	1/0	-	-	-	-	-	3/0	2/0	5/1	5/0
*M. daubentonii*	-	10/0	2/0	7/0	14/0	9/0	14/0	38/0	18/0	42/0	55/0
*M. emarginatus*	-	-	-	-	-	-	-	6/0	2/0	6/0	42/0
*M. myotis*	7/1	117/1	37/0	238/4	555/0	452/2	577/7	434/1	738/16	500/13	612/86
*M. mystacinus*	-	-	2/0	3/0	4/0	-	4/0	8/0	-	18/1	3/0
*M. nattereri*	-	3/0	6/0	3/0	5/0	7/0	2/0	15/0	28/0	25/1	20/0
*E. nilssonii*	-	3/0	-	12/0	4/0	5/0	6/0	11/1	7/0	4/0	10/1
*E. serotinus*	-	1/0	-	-	-	2/0	-	-	-	-	-
*P. pipistrellus*	-	-	-	-	-	-	2/0	-	-	-	437/0
*N. noctula*	-	-	-	-	-	-	-	-	-	-	2/0
*V. murinus*	-	-	-	-	1/0	-	-	1/0	-	-	-
*B. barbastellus*	-	148/0	-	5/0	8/0	11/0	12/0	122/0	55/0	205/0	22/0
*P. auritus*	-	4/0	2/0	7/0	15/0	4/0	8/0	9/0	12/0	16/0	26/0
*P. austriacus*	-	2/0	-	-	5/0	1/0	-	9/0	1/0	-	14/0

Photographs by T. Bartonička, L. Bufka, J. Červený, B. Lehotská, R. Lehotský, J. Matějů, J. Šafář, P. Tájek, J. Vogeltanz, and O. Vojtěch.

### Recent Presence of Geomycosis

**Figure 2:**
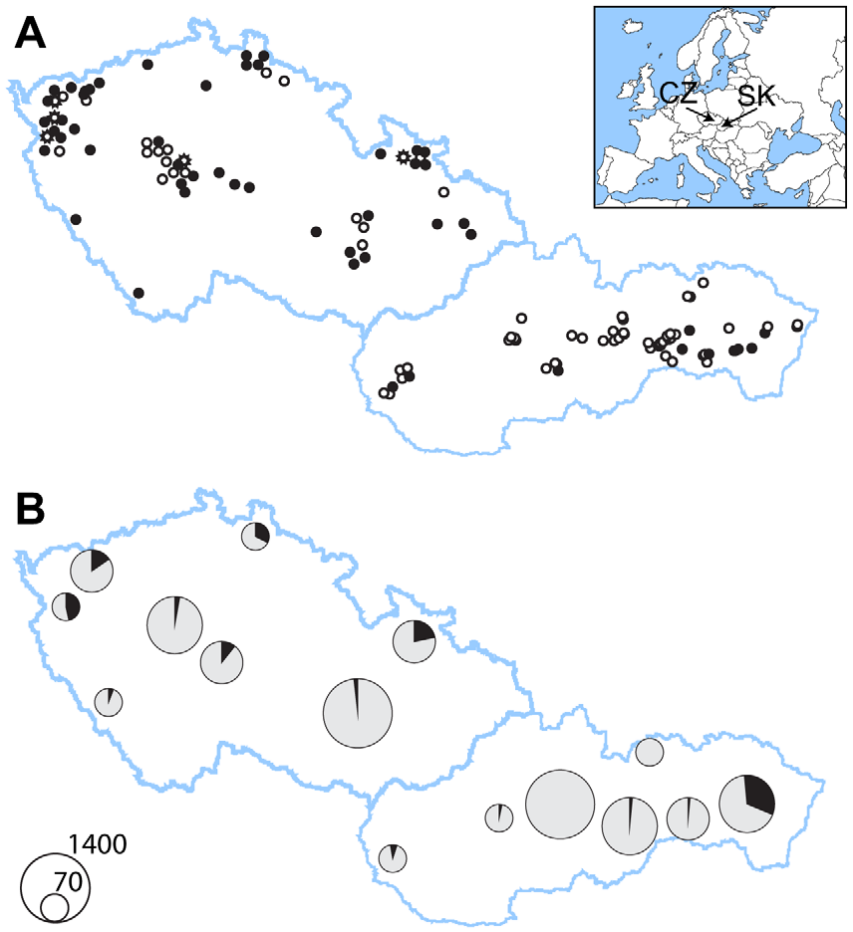
Occurrence of *Geomyces destructans* **in the Czech Republic and Slovakia.** (A) Distribution of WNS on the background of localities targeted for WNS screening. Some circles represent more than one hibernaculum. White circles -localities censused in 2009 and 2010; black circles - localities with WNS-suspect bats; stars - localities with photographic evidence of WNS in 2007 and 2008. (B) Prevalence of WNS-suspect individuals from *Myotis myotis* populations. Data pooled according to region; circle size is proportional to the population size.

Targeted on-site inspections of WNS-like clinical signs (white fungal growths on a bat, loss of sheen on wing membranes, emaciated forearms or the whole body if the hair was wet - worded as and regarded as ‘WNS-suspect’ throughout the remainder of this text) commenced in 2008/2009, as a part of the regular bat census. Bats exhibiting white fungal growths on their muzzle and/or wings were found at 7 sites. In total, 6 bat species were affected; *M. myotis* (24 individuals), *M. blythii* (1), *M. brandtii* (1), *M. dasycneme* (1), *M. emarginatus* (1) and *M. mystacinus* (1). During regular monitoring through the most recent winter of 2009/2010 (January/February), WNS-suspect bats were found at 33 sites out of over 800 hibernacula in the Czech Republic (CZ) and Slovakia (SK), combined. Additionally, 98 sites were inspected again in late February and March, and the fungus was then sampled for cultivation, microscopic, and genetic analyses. During that time period, the incidence of WNS-suspect bats increased to 76 localities across CZ and SK (Fig. 2). Most often the WNS-suspect bats were *M. myotis* (375 individuals), but also included *M. blythii* (19), *M. dasycneme* (2), *M. bechsteinii* (1), *M. mystacinus* (1), and *M. nattereri* (1). Specific regions differed in the prevalence of WNS-suspect bats. The highest levels of infestations were concentrated in submountain humid to mesic regions, where 11 to 100% of *M. myotis* were WNS-suspect. Infestation was less frequent in the hibernacula within mountainous zones (Šumava Mts.: 5% WNS-suspect *M. myotis*; the majority of SK localities: 0–5%), and also in limestone regions (Bohemian: 3%, Moravian: 2%, and Slovak: 3% karsts).

Four localities with WNS-suspect bats in Central Bohemia were visually checked every two weeks between late February and March 2010. We found decreasing percentages of individuals with fungal growth on muzzle and wings towards the end of their hibernation.

### Occurrence of *Geomyces destructans*

**Figure 3:**
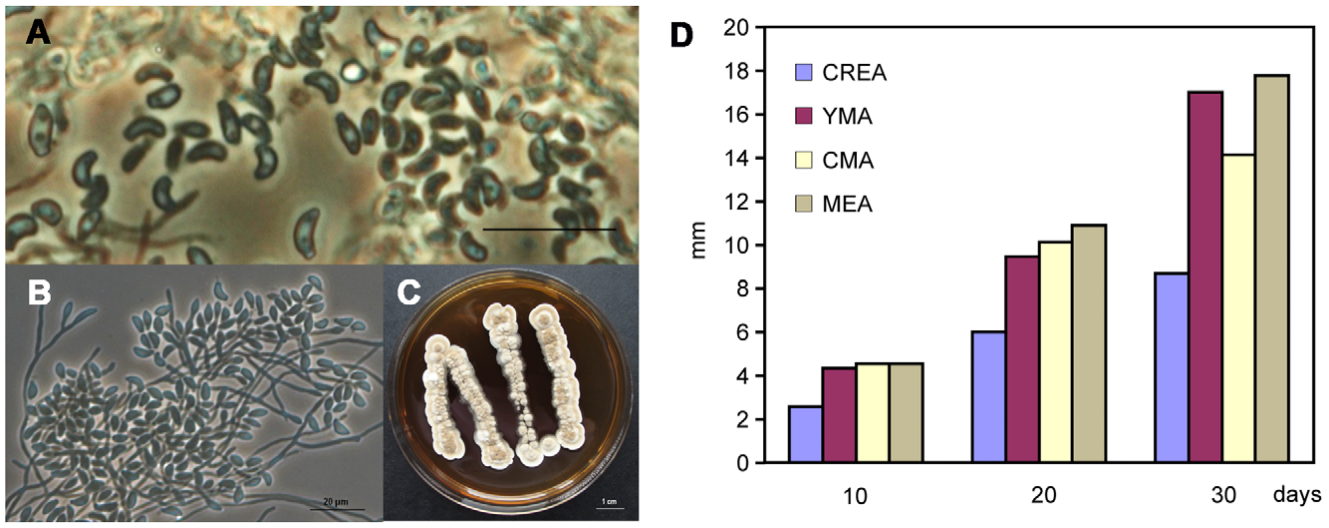
Spores and colonies of *G. destructans*. (A) Adhesive tape sample from the lesion of *M. myotis* photographed in Figure 1B, locality Malá Amerika Mines, Karlštejn, Czech Republic (Phase contrast). (B) *G. destructans* CCF3937. Conidiophores and arthroconidia (SDA, 14 days, 15°C, phase contrast). (C) Primary isolation of *G. destructans* CCF3942 (SDA, 1 month, 15°C). (D) Growth characteristics of *G. destructans* on four agar media at c. 15°C.

We collected the fungus on swabs and transparent adhesive tape between February 2, 2010 and March 26, 2010. In total, we collected the fungus from 90 bats, where 58 samples were collected onto cotton swabs, 10 onto nylon swabs, and 20 onto adhesive tape, one on both a nylon swab and adhesive tape and one sample consisted of shed hair (Table S1). Direct microscopic observation of the adhesive tape samples and nylon swabs from the WNS-suspect bats (*M. myotis*) confirmed the presence of conidia and mycelia with morphology consistent with *G. destructans* on 22 bats (Fig. 3A). Out of the 48 cultures, we isolated *G. destructans* from 16 (Table S1, Fig. 3B, C); and of these, 6 originated from the nylon swabs, 9 from the cotton swabs, and 1 from the adhesive tape sample. The isolates showed microscopic features typical of *G. destructans* (according to [3]), *i. e.* branched conidiophores with intercalary, lateral and terminal arthroconidia, conidia with a truncate base, mostly 5.8–7.7×2.7–3.4 µm, young conidia obovoid or cymbiform, mature conidia asymmetrical, crescent-like, curved (Fig. 3B). Colonies grow best on either malt extract or yeast and malt extract agars at 15°C (Fig. 3D). They grow slowly, reaching 18 mm after one month. The colonies were initially white, later pale brown to grey; the reverse uncoloured to brown or grey. These characteristics are similar to those previously described for isolates of *G. destructans* [3], [12].

We isolated DNA from 59 fungus samples, and 32 sequences, 933 base-pairs in length, were deposited into GenBank (Accession Numbers: HM584948 - HM584979; Table S1). Twenty-eight sequences were identical to previously sequenced *G. destructans* isolates [3], [9], [12]. Four other sequences, 3 from samples collected from *M. myotis*, and 1 from *M. bechsteinii*, exhibited a single A→G substitution in the sequenced region, namely, at position 144 of the internal transcribed spacer 1 gene (*ITS1*); additionally, one of those samples contained both the A and G alleles. Other samples did not amplify in the PCR reaction, or the sequences represented different taxa (Table S1).

At least 6 individuals were without an apparent mycelia cover, but had conspicuous lesions on either their auricles or wing membranes (Fig. 1B). *G. destructans* isolate CCF3942, was isolated from a sample taken from the lesion, and identified both by direct microscopy and cultivation (Fig. 3A).

### Population Size Trend of *Myotis myotis*

**Figure 4:**
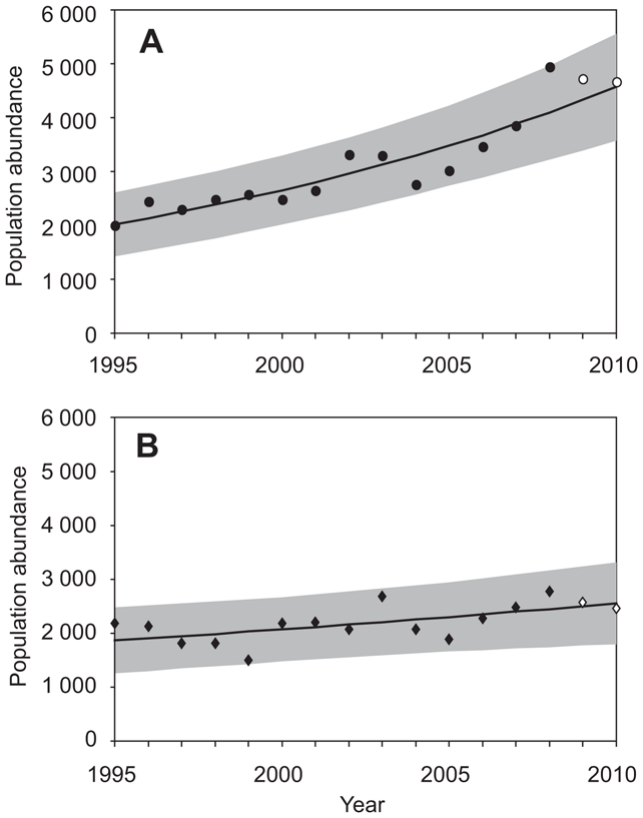
Upward population trends of hibernating *M. myotis*. In the Czech (A) and Slovak Republic (B) the trends were modelled over the period 1995–2010 by fitting Poisson regression allowing for over-dispersion in the data. The point prediction (solid line) and 95% prediction intervals (shaded area) are based on observations up to 2008 (solid symbols) and then extrapolated to 2009 and 2010. The open symbols represent observed data for 2009 and 2010.

Both the CZ and SK populations of *M. myotis* have been continuously growing during the studied period (Fig. 4). The average annual realized growth rate per capita of the CZ population is 0.058 (95% CI -0.008 to 0.122), corresponding to an increase of about 6% per year. In SK, the average annual growth rate is 0.008 (95% CI -0.087 to 0.103), corresponding to an increase of about 1% per year. Since 2008, the numbers of CZ and SK populations have declined by 6% and 11%, respectively (the joint numbers declined by 8%). However, the declining numbers fall well within the prediction intervals calculated for 2009 and 2010 (Fig. 4). Hence, there is as yet no evidence for a change in the population trend (CZ: *p* = 0.88, SK: *p* = 0.81). These conclusions remained unchanged after input of the missing data (CZ: *p* = 0.82, SK: *p* = 0.82).

## Discussion

We demonstrated that the fungus *G. destructans* is present in Central Europe, and that it is accompanied by aspects that might be suggestive of WNS (specifically white fungal growth on muzzle and wings, the skin lesions, loss of sheen on wing membranes, emaciated forearms or the whole body if the hair was wet). We have not conducted a histopathologic examination [4], [5], as no animals were euthanized in the course of this study; however, *G. destructans* was isolated from a scarred ear of a *M. myotis* individual without any apparent fungal growth. The presence of *G. destructans* has been previously demonstrated [12], [13], but the bat examined by Puechmaille et al. [12] was healthy, and Wibbelt et al. [13] reported a bat from Hungary with *G. destructans* growth to have survived until its next hibernation without any subsequent manifestation of the fungus. Neither study affirmed the presence of the disease, due to the absence of mass mortality in European bats; this contrasting with the disastrous population declines that have been seen in North America [1], [2]. We have shown that the *G. destructans* infection in our study exhibited a marked difference in the possible impacts on the bat populations compared to the North American case. Long-term population census data indicate an increase in population size in *M. myotis* in the Czech Republic and Slovakia, followed by a minor decline in 2009 and 2010, but well within the prediction interval for new data. Consequently, future observations are necessary in order to decide on the causality between possible WNS and bat population trends in Europe. An association of this population size fluctuation with the emergence of *G. destructans* infection cannot be ruled out, however, at the moment, we treat the result with caution. Our population trend analysis showed that the decline likely either represents a natural population fluctuation. Further monitoring will be necessary for a more complete evaluation of this trend.

The incidence of white fungal patches, a clinical sign of WNS, in hibernating bats in CZ and SK, increased markedly in 2010, suggestive of an epizootic spread of the fungus. Seasonally, more WNS-suspect bats were found late in their hibernation; although, the fungal growths disappeared prior to their leaving the hibernacula. This is in accord with previous information that *G. destructans* grows slowly, and that visually apparent mycelia mostly develop in the late winter and early spring [1], [9], [12]. Direct observations of arousing bats suggested that the infected bats tend to groom and remove surface mycelia immediately after arousal. According to our data, sampling the fungus onto nylon swabs enabled successful cultivations, even from lesions without visible mycelia. Previous studies have shown that isolations of *G. destructans* cultures were relatively rare, despite the presence of fungal spores in the samples that were revealed microscopically [9], [14]. Our results on a small sample size might help improve future sampling methodology to better facilitate the culture diagnostics of the pathogen.

Sequences of the *ITS1* gene showed for the first time to our knowledge polymorphism in the gene of *G. destructans*. In general, the *ITS* region has been used in WNS-related studies as a conservative marker that facilitates molecular identification of fungal species, similar in principle to DNA barcoding [1], [3], [9], [12], [13]. There are 33 sequences of the *G. destructans ITS* region in GenBank (retrieved on June 4, 2010), and all are identical. We have found four samples with a new allele. Genes encoding ribosomal RNA exhibit a low variability across large areas in fungi [15], [16], so we can speculate that occurrence of *G. destructans* in Europe predates its presence in North America, as was suggested by [13]. Our inspection of the photographed bats with fungal growths since 1995 further supports this assumption. If *G. destructans* was historically present in Europe, why has it never been detected on a large scale before (on the other hand, see [17])? During more than four decades of continuous monitoring in CZ, we have only detected faint fungal-like growths on hibernating bats since the 1990s. Our microscopic and genetic analyses showed that such a faint sheen might represent a wide spectrum of organisms, including nematodes. While some photographs might be debatable, we believe that Figure 1A shows an infection of *G. destructans*. In Javoříčské caves in north-eastern part of CZ, where the earliest photographic record of infected *M. myotis* originated, the species is recently rare. Later photographs from the north-western part of CZ coincide with regions with multiple positive records from the winter 2008/2009, as well as the highest infestation in 2010.

These facts indirectly support the hypothesis, presented above, that *G. destructans* was a resident element in Europe prior to its first appearance in North America [13]. If that is the case, why does WNS not, and why in the recent past did it not, cause mass mortality in Europe? At the moment, we lack the data that would answer these questions unequivocally, but we agree with the hypothesis of Wibbelt et al. [13] that differences in clustering behaviour in the most affected species (*M. lucifugus* vs. *M. myotis*) during hibernation might play an important role.

Until now, no other agent except *G. destructans* has been consistently associated with WNS [1], [3], [4], [5], [9], and we can further assume that the proximate effects of the fungus result in increased arousal frequency, flight activity in and outside of the hibernacula, and secondary infections. The mass mortality accompanying WNS is present in North America, but not in Europe. Different strategies of hibernation in the European underground hibernacula and those in North America could magnify the final effects of a yet undefined causality chain of *G. destructans* infection that leads to fatal consequences. While in Europe bats tend to hibernate isolated or to form small clusters, in North America, some hibernacula are characterized by very large aggregations of hibernating bats, amounting to thousands of individuals. Within such large clusters, multiple appearances of infected bats, their repeated arousals, grooming, and temperature increase would lead to the disturbance of neighbouring animals, potentially spreading across the cluster, as in a shock wave. In addition to the behavioural disturbances, large clusters would be influenced by density-dependent disease transmission [18]. Seen from an evolutionary perspective, WNS may act as a strong selection force that drives a change in hibernation strategy from hibernation in large clusters to a preference for less-populated hibernacula. This is the prevailing hibernation strategy in European *Myotis*. The hypothesis that this strategy was possibly selected for by previous mass mortality events, and the history of fungus-bat co-evolution [13] is indirectly supported by data on the historical occurrence of *M. bechsteinii*. In contrast to *M. myotis*, which first appeared in Central Europe in the Late Holocene, *M. bechsteinii* has been a constant element of the Mid-European interglacial communities since Early Pliocene. Mass accumulations of bat skeletal remains in European cave deposits of the Pleistocene and Pliocene age were often dominated by this species [19]. Currently, *M. bechsteinii* is a rare species that mostly avoids hibernation in caves and mines [20]. This suggests its regular hibernation in caves in the past with occasional mortality events. Assuming that some of the past mass mortality events in hibernacula could have been a result of a disease is not beyond the realm of possibility.

Unfortunately, the idea as to whether the disappearance of *M. bechsteinii* from caves was caused by recurring *G. destructans* infection, or a similar agent, is as yet merely speculation, and it might not be possible to reveal any hard facts supporting it. Nevertheless, the history of outburst events of *G. destructans*, environmental factors which could cause the outbursts, as well as the interactions between the fungus and hibernating bats are worthy of very detailed study. Further research of the ecological and genetic differentiation of hosts and pathogens might well provide crucial information for an assessment of the impacts of WNS (cf. [2]).

### Conclusions

We have shown that *Geomyces destructans*, the suspected infectious agent of WNS, is present across CZ and SK, without distinctive areas of prevalence. The reported incidence of its occurrence has increased since 2008, but it has likely been present since 1995, at the very least. To date, mass mortality has not been recorded, and the population fluctuation of *M. myotis* observed in 2009 and 2010 cannot be causally linked to the emergence of the disease. Nevertheless, we assume that white-nose syndrome is present in Europe. Future research should be aimed at establishing the precise effects of the disease on bats in Europe, as well as to elucidate the possible reasons for its less-severe impacts on the continent, whether it turns out to be immunological resistance, disparity in hibernating behaviour, genetic differences and associated virulence between European and American isolates of the pathogen, or environmental factors affecting the fungal growth.

## Materials and Methods

### Material

We used nylon swabs (microRheologics, Brescia, Italy), cotton swabs, or transparent adhesive tape to collect the 90 samples of fungi from the muzzle and wing membranes of hibernating bats. The nylon swabs were used according to the manufacturer's recommendations. The cotton swabs were placed directly into 1.5 ml plastic tubes as per [12], and the adhesive tape was stuck onto microscopic slides as per [13]. In total, we collected 10 samples using the nylon swabs, 58 samples using the cotton swabs, 20 using the adhesive tape, and 1 using both the nylon swab and tape. One other sample consisted of shed hair.

We examined photographs of hibernating bats, taken prior to 2009, for the presence of white fungal patches. The database consisted of photographs from 1994–1998 and from 2003–2010.

### Hibernacula Monitoring

The bat populations had been monitored in their underground hibernacula once a year, since 1969 [21]. The program currently consists of almost 900 sites [22], [23], [24]. In 2010, besides the standard census monitoring, 98 sites were repeatedly inspected in March. The animals were illuminated for a short time. The research adhered to the conditions of Permit #00356/KK/2008/AOPK for CZ, certificate of competency per Law No. 114/1992; for SK we employed Licence #2598/715/03-5.1pil, 5376/2009-2.1/jam, from the Ministry of Environment of the Slovak Republic, certificate of competency per Law No. 543/2002.

### Fungal Cultures

We conducted a mycological examination of 48 nylon swabs, cotton swabs and adhesive tape samples from the WNS-suspect bats, from 19 localities in CZ and SK (Table S1). Of those, 15 samples exhibited distinctive spores of *G. destructans* under direct microscopic observation of the nylon swabs and adhesive tapes (Fig. 1A). We inoculated the fungal material from the swabs and tapes onto Sabouraud dextrose agar plates (SDA, [25]) and incubated them in the dark at two temperatures (c. 7°C and 15°C). After 14 or more days, we isolated the outgrowing colonies of *G. destructans* and any other organisms. We identified the isolates according to [3], based on their phenotypic characteristics. Seven isolates are deposited at the Culture Collection of Fungi (CCF), Charles University in Prague, and 3 additional isolates in the Czech Collection of Microorganisms (CCM) at Masaryk University in Brno (Table S1).

To assess the basic growth characteristics, we studied three isolates of *G. destructans* (CCF 3937, 3938, 3939) at c. 15°C on four different agar media: malt extract agar (MEA), corn meal agar (CMA), yeast and malt extract agar (YMA), and creatine sucrose agar (CREA; [25]). We measured the colonies after 10, 20, and 30 days.

### DNA Sequencing

We isolated the fungal DNA directly from the 33 cotton swabs which were not used for the mycological examination, using a ZR Genomic DNA II kit (Zymo research, Orange, CA, USA), and 26 from adhesive tape and culture isolates, using a DNeasy Tissue Kit (Qiagen, Halden, Germany). In the initial screening, we amplified the genes encoding the partial SSU, the complete SSU intron, ITS1, 5.8S rRNA, ITS2, and the partial LSU, using universal fungal primers ITS-myko-F (5′-CAAACTTGGTCATTTAGAGGAA-3′) and ITS-myko-R (5′-CCTCCGCTTATTGATATGCT-3′). The PCR reactions, in a volume of 50 µl, consisted of 1× La buffer, 100 µm dNTPs, 50 pm of each primer, 1U LA DNA polymerase (Top-bio), and 2 µl DNA. Cycling conditions were 94°C for 5 min, followed by 30 cycles of 94°C for 20 s, 55°C for 20 s, and 68°C for 1 min. We purified the PCR products from an agarose electrophoresis gel using a Zmoclean gel DNA recovery kit (Zymo Research).

To increase the specificity of the amplification, we further utilised primers designed for *G. desctructans* found in France: ITS-F (5′-TCCTCCGCTTATTGATATGC-3′) and ITS-R (5′-GGAAGTAAAAGTCGTAACAAGG-3′) [12]. PCR reactions consisted of 1× Buffer, 100 µm dNTPs, 3 mm MgCl_2_, 25 µm of each primer, 1 U Platinum *Taq* (Invitrogen, Carlsbad, CA, USA), and 2 µl DNA. Cycling conditions followed the touch-down protocol of [12]. The PCR reaction yielded single bands that were purified using PCR Purification Kit (Qiagen). All products were commercially sequenced from both directions, using BigDye® Terminator sequencing chemistry (Applied Biosystems, Foster City, CA, USA) on 3100-Avant Genetic Analyzer (Applied Biosystems) sequencers.

### Data Analyses

We assembled the contigs in Aligner 3.5.6 (CodonCode, Dedham, MA, USA), and we identified the resulting sequences by comparing them to GenBank, using BLASTN 2.2 [26].

For the population trend analyses, we selected 106 hibernacula, with the most complete continuous records since 1995. The average annual realized growth rate per capita was estimated by regression through the origin, according to [27]. To test the hypothesis of significant changes in population size, we used data collected over years 1995 to 2008 from 106 hibernacula, and extrapolated the time trends to 2009 and 2010. We computed the prediction intervals, considering both the uncertainty about future count expectation and the random error of Poisson-distributed observations [28]. We included an estimation of the dispersion parameter to address the unexplained extra-Poisson variance. The model fitting and prediction was performed using Stata/IC 10.1 statistical software (StataCorp, College Station, TX, USA). To test the effect of the missing data, we reanalysed the dataset with the missing values input as a combination of the last observation carried forward and the next observation carried backward methods.

## Supporting Information

Table S1 Material examined for *Geomyces destructans* presence found in the Czech Republic and Slovakia, host species, localities, direct microscopic examination, sequence Accession Numbers, and isolate numbers.
(0.13 MB PDF)
