# Supplementary material for: Increasing Incidence of Geomyces destructans Fungus in Bats from the Czech Republic and Slovakia
Source: PLoS One. 2010 Nov 5;5(11):e13853. doi: 10.1371/journal.pone.0013853 (PMC2974652; doi:10.1371/journal.pone.0013853)
Supplement: Table S1 — Material examined for Geomyces destructans presence found in the Czech Republic and Slovakia, host species, localities, direct microscopic examination, sequence Accession Numbers, and isolate numbers. (0.13 MB PDF) [file pone.0013853.s001.pdf]

Supporting Information for: Increasing Incidence of *Geomyces destructans* Fungus in Bats from the Czech Republic and Slovakia

Natália Martínková*, Peter Bačkor, Tomáš Bartonička, Pavla Blažková, Jaroslav Červený, Lukáš Falteisek, Jiří Gaisler, Vladimír Hanzal, Daniel Horáček, Zdeněk Hubálek, Helena Jahelková, Miroslav Kolařík, Ľuboš Korytár, Alena Kubátová, Blanka Lehotská, Roman Lehotský, Radek K. Lučan, Ondřej Májek, Jan Matějů, Zdeněk Řehák, Jiří Šafář, Přemysl Tájek, Emil Tkadlec, Marcel Uhrin, Josef Wagner, Dita Weinfurtová, Jan Zima, Jan Zukal, Ivan Horáček

* Corresponding Author E-mail: martinkova@ivb.cz

Table S1. Material examined for *Geomyces destructans* presence found in the Czech Republic and Slovakia, host species, localities, direct microscopic examination, sequence Accession Numbers, and isolate numbers.

Number	Host species	Locality	Geographic coordinates	Collection date	Direct microscopy	Accession Number	Cultivation
1[t]	*Myotis myotis*	Pod Medveďou skalou, Modra, SK	48.3748, 17.3	5.3.2010	neg.	neg.	neg.
2[t]	*M. myotis*	Pod Medveďou skalou, Modra, SK	48.3748, 17.3	5.3.2010	neg.	neg.	neg.
SK10[c]	*M. myotis*	Banská Štiavnica, mines, SK	48.47, 18.9	3.3.2010	n/a	n/a	neg.
SK9[c]	*M. myotis*	Banská Štiavnica, mines, SK	48.47, 18.9	3.3.2010	n/a	n/a	neg.
SK1[c]	*M. myotis*	Dubník Mines, SK	48.9, 21.45	1.3.2010	n/a	n/a	neg.
SK2[c]	*M. myotis*	Dubník Mines, SK	48.9, 21.45	1.3.2010	n/a	n/a	neg.
SK3[c]	*M. myotis*	Dubník Mines, SK	48.9, 21.45	1.3.2010	n/a	n/a	neg.
SK4[c]	*M. myotis*	Dubník Mines, SK	48.9, 21.45	1.3.2010	n/a	n/a	neg.
SK5[c]	*M. blythii*	Dubník Mines, SK	48.9, 21.45	1.3.2010	n/a	n/a	neg.
SK6[c]	*M. myotis*	Dubník Mines, SK	48.9, 21.45	2.3.2010	n/a	HM584979	CCM8378
SK7[c]	*M. blythii*+*M. dasycneme*	Dubník Mines, SK	48.9, 21.45	2.3.2010	n/a	(uncultured fungus)	neg.
SK8[c]	*M. daubentonii*	Dubník Mines, SK	48.9, 21.45	2.3.2010	n/a	n/a	neg.
6[t]	*M. myotis*	Nová Drátenická Cave, CZ	49.2912, 16.7279	6.3.2010	*G.d.*	HM584948	n/a
7[t]	*M. myotis*	Nová Drátenická Cave, CZ	49.2912, 16.7279	6.3.2010	neg.	neg.	n/a
5[t]	*M. myotis*	Stará Drátenická Cave, CZ	49.2912, 16.728	6.3.2010	*G.d.*	neg.	n/a
10[t]	*M. myotis*	Byčí skála Cave, CZ	49.3084, 16.6923	6.3.2010	*G.d.*	neg.	n/a
11[t]	*M. myotis*	Byčí skála Cave, CZ	49.3084, 16.6923	6.3.2010	*G.d.*	HM584950	n/a
20[t]	*M. dasycneme*	Byčí skála Cave, CZ	49.3084, 16.6923	6.3.2010	neg.	neg.	n/a
8[t]	*M. myotis*	Jestřábka Cave, CZ	49.3087, 16.6744	6.3.2010	*G.d.*	HM584949	n/a
9[t]	*M. myotis*	Jestřábka Cave, CZ	49.3087, 16.6744	6.3.2010	*G.d.*	(Collembola)	neg.

21[c]	*M. myotis*	Diana Mine, Dolní Loučky, CZ	49.3558, 16.3505	21.3.2010	n/a	n/a	*G.d.*
22[c]	*M. myotis*	Diana Mine, Dolní Loučky, CZ	49.3558, 16.3505	21.3.2010	n/a	n/a	*G.d.*
15[t]	*M. myotis*	Králova Cave, CZ	49.3591, 16.4153	18.3.2010	neg.	(Nematoda)	neg.
16[t]	*M. myotis*	Králova Cave, CZ	49.3591, 16.4153	18.3.2010	*G.d.*	neg.	neg.
17[t]	*M. myotis*	Králova Cave, CZ	49.3591, 16.4153	18.3.2010	*G.d.*	(Basidiomycota)	neg.
18[t]	*M. myotis*	Králova Cave, CZ	49.3591, 16.4153	18.3.2010	*G.d.*	neg.	n/a
19[t]	*M. myotis*	Králova Cave, CZ	49.3591, 16.4153	18.3.2010	*G.d.*	HM584952	n/a
12[t]	*M. myotis*	Kateřinská Cave, CZ	49.3611, 16.7095	18.3.2010	*G.d.*	neg.	neg.
13[t]	*M. myotis*	Kateřinská Cave, CZ	49.3611, 16.7095	18.3.2010	neg.	(Nematoda)	neg.
14[t]	*M. myotis*	Kateřinská Cave, CZ	49.3611, 16.7095	18.3.2010	*G.d.*	HM584951	neg.
GD28[c]	*M. myotis*	Černá řeka Mine, CZ	49.4196, 12.7352	7.3.2009	n/a	(Basidiomycota)	n/a
N1[n]	*M. myotis*	Solenice, CZ	49.6148, 14.1946	5.3.2010	*G.d.*	HM584954	CCF3938
N2[n]	*M. myotis*	Solenice, CZ	49.6148, 14.1946	5.3.2010	*G.d.*	HM584955	CCF3939
N21[n,t]	*Rhinolophus hipposideros* (dead)	Javořîčske Caves, CZ	49.6694, 16.9131	25.3.2010	*G.d.*	n/a	neg.
23[c]	*M. myotis*	Panny Marie Sněžné Mine, Hrubá voda, CZ	49.6713, 17.4173	19.3.2010	n/a	n/a	neg.
24[c]	*M. myotis*	Panny Marie Sněžné Mine, Hrubá voda, CZ	49.6713, 17.4173	19.3.2010	n/a	n/a	neg.
25[c]	*M. myotis*	Velká Střelná, Libavá, CZ	49.7, 17.53	19.3.2010	n/a	n/a	*G.d.*
26[c]	*M. myotis*	Velká Střelná, Libavá, CZ	49.7, 17.53	19.3.2010	n/a	n/a	*G.d.*
27[c]	*M. myotis*	Velká Střelná, Libavá, CZ	49.7, 17.53	19.3.2010	n/a	n/a	neg.
GD19[c]	*M. myotis*	Sv. Jan na poušti Mine, CZ	49.7396, 12.966	21.3.2010	n/a	HM584966	n/a
N15[n]	*M. myotis*	Sv. Jan na poušti Mine, CZ	49.7396, 12.966	26.2.2010	*G.d.*	HM584957	CCF3943
GD7[c]	*M. myotis*	Sv. Anna Mine, Výškov, CZ	49.7491, 13.0026	23.2.2010	n/a	HM584960	n/a
N8[n]	*M. myotis*	Líšnice, mine, CZ	49.7607, 16.8629	16.3.2010	*G.d.*	n/a	neg.
N19[hair]	shed hair	Nový Knín, CZ	49.7892, 14.2945	17.3.2010	neg.	n/a	neg.
N16[n]	*R. hipposideros*	Nový Knín, CZ	49.7892, 14.2945	17.3.2010	neg.	n/a	neg.
N17[n]	*M. myotis*	Nový Knín, CZ	49.7892, 14.2945	17.3.2010	*G.d.*	HM584958	CCF3944
N18[n]	*M. myotis*	Nový Knín, CZ	49.7892, 14.2945	17.3.2010	neg.	n/a	neg.
GD35[c]	*M. myotis*	Český Šternberk, CZ	49.8097, 14.9266		n/a	HM584978	n/a
GD8[c]	*M. myotis*	Trdlina, mine, CZ	49.8832, 12.8133	14.2.2010	n/a	(Ascomycota)	n/a
GD30[c]	*M. myotis*	Věra Mine, CZ	49.9159, 12.7767	16.3.2010	n/a	HM584973	n/a

GD9[c]	*M. bechsteinii*	Lazurový vrch, mine near castle, CZ	49.9164, 12.7772	5.3.2010	n/a	HM584961	n/a
GD31[c]	*M. myotis*	Liblín, house cellar, CZ	49.9171, 13.5395	14.3.2010	n/a	HM584974	n/a
GD29[c]	*M. myotis*	Jeskyně Inků Mine, CZ	49.9174, 12.7676	26.3.2010	n/a	HM584971	n/a
GD6[c]	*M. myotis*	Pístov, house cellar, CZ	49.9229, 12.7653	18.2.2010	n/a	neg.	n/a
GD1[c]	*M. myotis*	Malá Amerika Mines, CZ	49.9566, 14.1901	22.2.2010	n/a	HM584959	n/a
GD32[c]	*M. myotis*	Malá Amerika Mines, CZ	49.9566, 14.1901	22.2.2010	n/a	HM584975	n/a
N0[n]	*M. myotis*	Malá Amerika Mines, CZ	49.9566, 14.1901	2.2.2010	n/a	HM584953	CCF3937
N10[n]	*M. myotis*	Malá Amerika Mines, CZ	49.9566, 14.1901	16.3.2010	*G.d.*	n/a	neg.
N12[n]	*M. myotis*	Malá Amerika Mines, CZ	49.9566, 14.1901	16.3.2010	*G.d.*	HM584956	CCF3941
N11[t]	*M. myotis*	Malá Amerika Mines, CZ	49.9566, 14.1901	16.3.2010	*G.d.*	n/a	neg.
N13[t]	*M. myotis*	Malá Amerika Mines, CZ	49.9566, 14.1901	16.3.2010	*G.d.*	n/a	CCF3942
GD26[c]	*M. myotis*	Nečtiny, house cellar, CZ	49.9609, 13.1615	12.3.2010	n/a	HM584971	n/a
GD27[c]	*Barbastella barbastellus*	Nečtiny, house cellar, CZ	49.9609, 13.1615	12.3.2010	n/a	neg.	n/a
34[c]	*M. myotis*	Franz Mine, Moravice, CZ	49.9774, 17.2829	6.3.2010	n/a	n/a	CCM8380
35[c]	*M. myotis*	Franz Mine, Moravice, CZ	49.9774, 17.2829	6.3.2010	n/a	n/a	*G.d.*
32[c]	*M. myotis*	Mařka Mine, Bohdíkov, CZ	50.0215, 16.8911	9.3.2010	n/a	n/a	neg.
33[c]	*M. myotis*	Mařka Mine, Bohdíkov, CZ	50.0215, 16.8911	9.3.2010	n/a	n/a	neg.
GD20[c]	*M. myotis*	Manganka, CZ	50.055, 12.716	10.3.2010	n/a	HM584967	n/a
GD21[c]	*M. myotis*	Manganka, CZ	50.055, 12.716	10.3.2010	n/a	HM584968	n/a
GD22[c]	*M. nattereri*	Manganka, CZ	50.055, 12.716	10.3.2010	n/a	HM584969	n/a
GD24[c]	*M. myotis*	Staré Sedlo, Hrušková, mine, CZ	50.1679, 12.7098	18.3.2010	n/a	neg.	n/a
28[c]	*M. myotis*	Rejvíz, mine I a II, CZ	50.219, 17.3247	20.3.2010	n/a	n/a	neg.
29[c]	*M. myotis*	Rejvíz, mine I a II, CZ	50.219, 17.3247	20.3.2010	n/a	n/a	*G.d.*
30[c]	*M. myotis*	Rejvíz, mine I a II, CZ	50.219, 17.3247	20.3.2010	n/a	n/a	CCM8379
31[c]	*M. myotis*	Jeskyně na Špičáku Cave, Supíkovice, CZ	50.2833, 17.25	18.3.2010	n/a	n/a	neg.
GD3[c]	*M. myotis*	Dolní Studenec, CZ	50.3, 12.53	24.2.2010	n/a	neg.	n/a
GD2[c]	*M. myotis*	Nejdek, CZ	50.32, 12.73	23.2.2010	n/a	(uncultured fungus)	n/a
GD23[c]	*M. myotis*	Červená štola (Pernink), CZ	50.3568, 12.7979	7.3.2010	n/a	HM584970	n/a
GD15[c]	*M. myotis*	Mauritius Mine, CZ	50.3914, 12.8333	6.3.2010	n/a	HM584965	n/a
GD16[c]	*M. mystacinus*	Mauritius Mine, CZ	50.3914, 12.8333	6.3.2010	n/a	(Basidiomycota)	n/a
GD17[c]	*M. brandtii*	Mauritius Mine, CZ	50.3914, 12.8333	6.3.2010	n/a	neg.	n/a

GD18[c]	*Plecotus auritus*	Mauritius Mine, CZ	50.3914, 12.8333	6.3.2010	n/a	neg. (uncultured fungus)	n/a
GD4[c]	*Eptesicus nilsoni*	Wildbahner, CZ	50.3923, 12.8216	28.2.2010	n/a		n/a
GD5[c]	*Plecotus sp.*	Wildbahner, CZ	50.3923, 12.8216	28.2.2010	n/a	neg.	n/a
GD33[c]	*M. myotis*	Schůdky Mine, Potůčky, CZ	50.42, 12.75		n/a	HM584976	n/a
GD34[c]	*M. myotis*	Ementál Mine, CZ	50.4328, 12.8339	9.2.2010	n/a	HM584977	n/a
GD14[c]	*M. mystacinus*	Horní Perníková Mine, CZ	50.4358, 12.8603	26.3.2010	n/a	HM584964	n/a
GD11[c]	*M. myotis*	Kohlreuter, CZ	50.4368, 12.8614	26.3.2010	n/a	HM584962	n/a
GD12[c]	*M. myotis*	Kohlreuter, CZ	50.4368, 12.8614	26.3.2010	n/a	HM584963	n/a
GD13[c]	*M. mystacinus*	Kohlreuter, CZ	50.4368, 12.8614	26.3.2010	n/a	neg.	n/a

c – cotton swab, n – nylon swab, t – adhesive tape sample; CZ – Czech Republic, SK – Slovakia; n/a – not available, *G.d.* – presence of conidia and mycelia with morphology consistent with *G. destructans*, neg. – negative for *G.d.*; taxa in parentheses – top blastn hits of DNA sequences other than *G. destructans*.
